# RNA-Seq for gene identification and transcript profiling of three *Stevia rebaudiana* genotypes

**DOI:** 10.1186/1471-2164-15-571

**Published:** 2014-07-07

**Authors:** Junwen Chen, Kai Hou, Peng Qin, Hongchang Liu, Bin Yi, Wenting Yang, Wei Wu

**Affiliations:** Agronomy College of Sichuan Agricultural University, Wenjiang, Chengdu, Sichuan 611130 China; Rice Research Institute of Sichuan Agricultural University, Wenjiang, Chengdu, Sichuan 611130 China; Agronomy College of Guizhou University, Guiyang Huaxi, Guizhou 550025 China (HCL); Agricultural Bureau of Leshan, Sichuan, 614000 China (WTY)

**Keywords:** Stevia rebaudiana, Transcriptome, RNA-seq

## Abstract

**Background:**

*Stevia* (*Stevia rebaudiana*) is an important medicinal plant that yields diterpenoid steviol glycosides (SGs). SGs are currently used in the preparation of medicines, food products and neutraceuticals because of its sweetening property (zero calories and about 300 times sweeter than sugar). Recently, some progress has been made in understanding the biosynthesis of SGs in *Stevia*, but little is known about the molecular mechanisms underlying this process. Additionally, the genomics of *Stevia*, a non-model species, remains uncharacterized. The recent advent of RNA-Seq, a next generation sequencing technology, provides an opportunity to expand the identification of *Stevia* genes through in-depth transcript profiling.

**Results:**

We present a comprehensive landscape of the transcriptome profiles of three genotypes of *Stevia* with divergent SG compositions characterized using RNA-seq. 191,590,282 high-quality reads were generated and then assembled into 171,837 transcripts with an average sequence length of 969 base pairs. A total of 80,160 unigenes were annotated, and 14,211 of the unique sequences were assigned to specific metabolic pathways by the Kyoto Encyclopedia of Genes and Genomes. Gene sequences of all enzymes known to be involved in SG synthesis were examined. A total of 143 UDP-glucosyltransferase (UGT) unigenes were identified, some of which might be involved in SG biosynthesis. The expression patterns of eight of these genes were further confirmed by RT-QPCR.

**Conclusion:**

RNA-seq analysis identified candidate genes encoding enzymes responsible for the biosynthesis of SGs in *Stevia,* a non-model plant without a reference genome. The transcriptome data from this study yielded new insights into the process of SG accumulation in *Stevia*. Our results demonstrate that RNA-Seq can be successfully used for gene identification and transcript profiling in a non-model species.

**Electronic supplementary material:**

The online version of this article (doi:10.1186/1471-2164-15-571) contains supplementary material, which is available to authorized users.

## Background

*Stevia* (*Stevia rebaudiana* Bertoni (2n = 22, Asteraceae)) is an important medical plant that is native to South America [1]. Among 230 species of this genus, only two *Stevia* species (*S. rebaudiana* and *S. phlebophylla*) can produce SGs (steviol glycosides) [2]. SGs are non-caloric and non-cariogenic sweeteners with functional and sensory properties superior to many other high-potency sweeteners [3, 4]. Because of their sweetening property (zero calories and about 300 times sweeter than sugar), SGs have been used widely in the preparation of medicines, beverages and neutraceuticals [5]. In general, SGs accumulate in *Stevia* leaves. Depending on the genotype and the growing and harvesting conditions, the concentration varies from 10% to 20% of the dry weight of leaves [6]. Currently, *Stevia* is a major source of natural SGs, which have received increasingly greater interest among different research fields [7].

The SG produced by *Stevia* is a mixture of at least eight different types, including ST (stevioside), RA-RF (rebaudiosides A-F), rubusoside and dulcoside A, a new diterpene glycoside recently isolated from *Stevia* [8]. Some progress has been made in understanding the biosynthetic pathway of SGs, including the identification of 17 steps catalyzed by different enzymes. The initial seven steps are similar to steps within the MEP (methyl erythritol-4-phosphate) pathway, catalyzing pyruvate to isopentenyl diphosphate. Several genes have been demonstrated to be involved in the first seven steps, including *DXS*, *DXR*, *MCT*, *CMK*, *MDS*, *HDS*, *HDR* and *IDI* [2]. The GA (gibberellic acid) biosynthetic pathway (from isopentenyl diphosphate to kaurenoic acid) is involved in the next five pathways, in which 4 genes (*GGDPS*, *CPPS*, *KS* and *KO*) are thought to participate. The last five steps, from kaurenoic acid to RA, are specific to the SG biosynthetic pathway [2]. Four genes, including *GGDPS*, *CPPS*, *KS* and *KO* are involved in the last five steps. Additionally, P450-dependent monooxygenases and glycosyltransferases are assumed to be involved in the modification of the triterpenoid backbone [9], and UGTs (UDP-glycosyltransferases), including *UGT85C2*, *UGT74G1* and *UGT76G1*, are also proposed to participate in the RA biosynthetic pathway [10]. Although the biosynthetic pathway of SGs has been extensively studied, the mechanism of biosynthesis and the genes involved in the pathway remain poorly understood.

SGs are the sweetest known natural sweeteners, but the taste perception of different SGs is strong depending on their patterns of glycosylation [11]. Among different SGs, RA has a much better taste perception than ST, which is being applied as a substitute for saccharose and is used in the treatment of diabetes mellitus, obesity, and hypertension [7]. Therefore, RA is a good replacement of ST [12]. Many plant culture practices aiming to increase the leaf yield and RA content have been studied [13]. However, molecular biology techniques have been scarcely used in the improvement of SG accumulation in *Stevia*, mostly due to the absence of available sequence information. Currently, there are only 160 sequences originating from *Stevia* that are available in the National Center for Biotechnology Information (NCBI) database. For this reason, it is difficult to isolate functional genes that govern important quality and agronomic traits of *Stevia*.

RNA-Seq, based on next-generation sequencing technology, is emerging as an attractive approach to understand transcriptome profiling. RNA-Seq provides a far more precise measurement of transcripts than other methods and has been successfully used for annotation, transcript profiling and/or SNP discovery in a number of plant species [14–17]. Furthermore, unlike microarrays, RNA-Seq does not require prior knowledge of gene sequences. In this study, we used Illumina RNA-Seq technology for identifying genes associated with SG biosynthesis in three *Stevia* geneotypes with different RA and ST contents. In total, 191,590,282 high-quality reads were generated, and 80,160 unigenes were obtained by *de novo* assembly. A total of 10,070 SSRs and 44,510 SNPs were also identified, which might be useful for *Stevia* molecular research. We also identified 636,2,464 and 2,041 unigenes with differential expression levels in SR-1, SR-2, and SR-3, as well as homologs of several unigenes involved in the SG biosynthetic pathway. Our study provides a platform of sequence information for global discovery of novel functional genes involved in the biosynthesis of SGs and demonstrates the powerful ability of high-throughput sequencing to identify candidate genes involved in novel metabolic pathways in non-model plant systems.

## Results and discussions

### The leaves of three *Stevia*genotypes (SR-1, SR-2 and SR-3) have dramatically different amounts of ST and RA

To select different genotypes of *Stevia* for comprehensive characterization of genes associated with SG (especially ST and RA) biosynthesis, we used HPLC to analyze the ST and RA contents in the leaves from three *Stevia* genotypes (SR-1, SR-2 and SR-3). SR-1 had relatively higher RA, with 2.19% ST and 6.91% RA, whereas SR-2 had a preponderance of ST (12.87% ST and 0.02% RA). RA was the most highly accumulated in SR-3 (9.35%), with the lowest amount of ST (1.23%) (Table 1). Based on the dramatic differences in the amounts of RA and ST, we assessed the gene expressions in all three genotypes to provide a more comprehensive overview of SG-associated gene profiles in plants with different SG expression patterns.

**Table 1:** **Comparison of ST and RA contents in leaves of chemical types SR-1, SR-2 and SR-3**

Sample	ST%	RA%
SR-1	2.19	6.91
SR-2	12.87	0.02
SR-3	1.23	9.35

### RNA-sequencing and *de novo*assembly of three genotypes of *Stevia*

To comprehensively survey the genes associated with SG formation and accumulation, we performed RNA-seq for SR-1, SR-2 and SR-3. RNA was extracted from the leaves of the three samples at the bud stage and used to develop cDNA libraries. In total, 61,710,194, 68,652,614 and 61,227,474 reads were achieved in the SR-1, SR-2, and SR-3 libraries, respectively (Table 2). To ensure the reliability of the libraries, we performed quality controls and obtained 60,113,164, 66,869,210 and 58,857,260 clean reads for SR-1, SR-2, SR-3. Due to the absence of reference genomic sequences, *de novo* assembly was applied to construct transcripts from these RNA-seq reads. In this study, we used Trinity (version: v2012-10-05) software [18] for *de novo* assembly of the Illumina reads, which has been demonstrated to be efficient for *de novo* reconstruction of transcriptomes from RNA-Seq data [18–20]. The reads from the three genotypes were pooled together for more comprehensive reconstruction of transcripts, and a total of 171,837 contigs were obtained from the clean reads of the pool with a mean length of 969 bp and length ranging from 201 bp to 15,537 bp (Table 3). Among the 171,837 contigs, 80,160 unigenes were obtained.

**Table 2:** **Assessment of assembly quality for** ***Stevia*** **libraries of three different genotypes**

	SR-1	SR-2	SR-3
Raw reads	61710194	68652614	61227474
Clean reads	60113164	66869210	58857260
Error (%)	0.03	0.03	0.03

**Table 3:** **Summary of assembly quality for** ***Stevia*** **RNA-seq**

Assembly quality parameters	
Contigs generated	171,837
Maximum contig length	15,537
Minimum contig length	201
Average contig length	969
Contigs 200–500 bp	71,627
Contigs 500-1 kb	40,288
Contigs 1-2 Kb	39,651
Contigs ≥ 2 Kb	20,271
N50 value	1,547

### Gene annotation

Unigenes annotation was performed by BLAST searching (E-value ≤10^-5^) against the Nr (NCBI non-redundant protein sequences), Nt (NCBI nucleotide sequences), Pfam (protein family), KOG (euKaryotic Ortholog Groups), Swiss-Prot (A manually annotated and reviewed protein sequence database), KEGG (Kyoto Encyclopedia of Genes and Genomes) and GO (Gene Ontology) databases [21]. A total of 41,946 unigenes (52.32% of all unigenes) were annotated with a significant BLAST result in the Nr database; 30,169 unigenes (37.63% of all unigenes) were annotated in Swiss-Prot database; and 32,074 unigenes were annotated in both the Pfam and GO databases. In total, 47,165 unigenes were annotated in the seven databases (Table 4).

**Table 4:** **Gene annotation by searching against public databases**

	Number of unigenes	Percentage (%)
Annotated in NR	41946	52.32
Annotated in NT	14292	17.82
Annotated in KEGG	14211	17.72
Annotated in SwissProt	30169	37.63
Annotated in PFAM	32074	40.01
Annotated in GO	32074	40.01
Annotated in KOG	19146	23.88
Annotated in all databases	47165	58.83
Total unigenes	80160	

### Gene ontology (GO) classification

**Figure 1:**
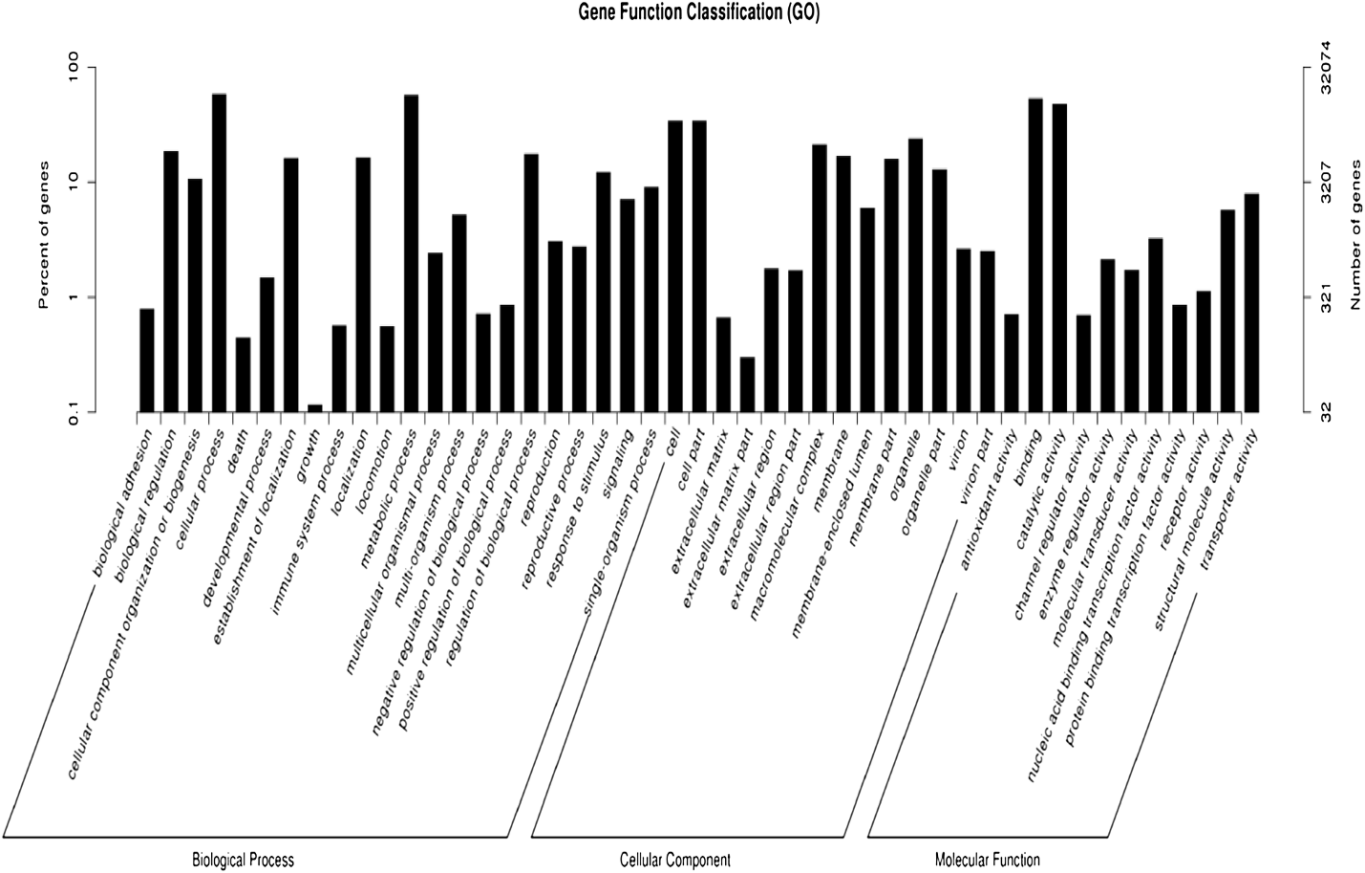
**Histogram of gene ontology classification.** The results are summarized in three main categories: biological process, cellular component and molecular function.

GO assignments were used to predict the functions of *Stevia* unigenes by classifying them into various biological processes [22]. Based on sequence homology, the 32,074 unigenes annotated in the GO database were categorized into 47 functional groups. Among these groups, “cellular process” and “metabolic process” were dominant within the “biological process” category, the “cell” and “cell part” categories were dominant in the “cellular component” category, and “binding” and “catalytic activity” were dominant in the molecular function category (Figure 1). Additionally, we noted that many genes were classified into the “biological regulation”, “organelle” and “catalytic activity” categories, whereas a few genes were classified into the “growth” and “extracellular matrix part” groups (Figure 1, and Additional file 1).

*Stevia* SGs are considered metabolic products and are glucosylated derivatives. Therefore, we hypothesized that the 18,292 unigenes classified into the “metabolic process” group and the 15,272 unigenes classified into the “catalytic activity” group might serve as good candidates for the identification of novel genes that participated in the SG biosynthesis and accumulation pathways (Figure 1, and Additional file 1).

### Functional classification by KEGG

**Figure 2:**
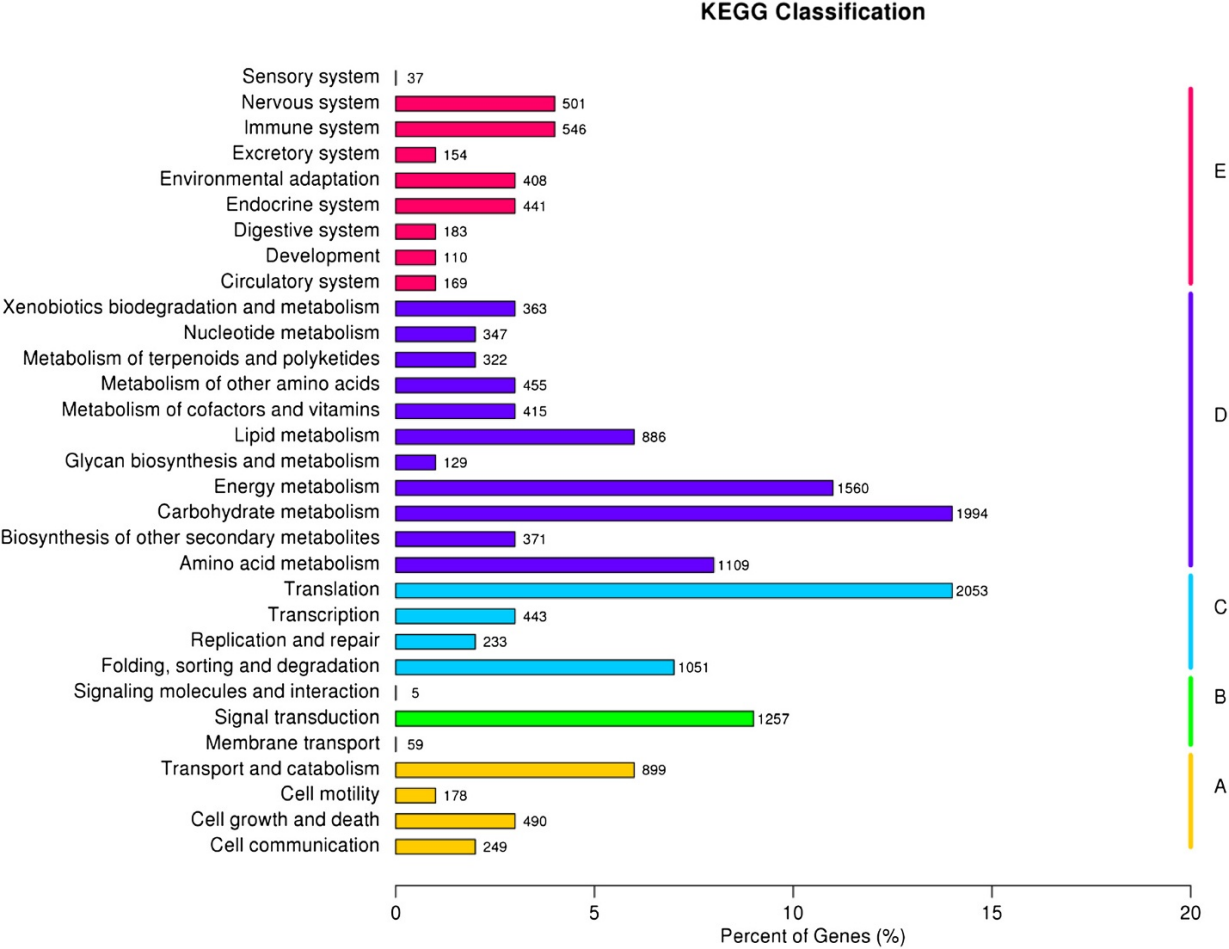
**Pathway assignment based on KEGG. (A)** Cellular Processes; **(B)** Environmental Information Processing; **(C)** Genetic Information Processing; **(D)** Metabolism; **(E)** Organismal Systems.

KEGG is thought to provide a basic platform for systematic analysis of gene function in terms of the networks of gene products [23]. To further identify the biological pathways that are active in *Stevia*, the 14,211 unigenes annotated by blast analysis against KAAS (KEGG Automatic Annotation Server) were mapped to 250 reference canonical pathways, and these pathways were classified into five main categories: “cellular processes”, “environmental information processing”, “genetic information processing”, “metabolism” and “organismal systems”. The pathways with most representation were “translation” (2,053 unigenes, 14.45%) and “carbohydrate metabolism” (1,994 unigenes, 14.01%) (Figure 2, Additional file 2). These annotations and classifications provided a resource for investigating specific pathways in *Stevia*, such as the SG biosynthetic pathway. SGs are tetracyclic diterpene glycosides; therefore, the 322 unigenes clustered into “metabolism of terpenoids and polyketides” might potentially be involved in the biosynthesis and metabolism of SGs. Among the 322 unigenes, 76 unigenes (23.53%) and 30 unigenes (9.32%) were classified into the “terpenoid backbone” and “diterpenoid biosynthesis” sub-pathways, respectively, and thus were more likely to be involved in SG biosynthesis for *Stevia* (Additional file 2).

### SSR and SNP detection

Molecular markers are extremely important for molecular biology research (e.g., gene mapping) and molecular breeding [24, 25]. We sought to identify candidate markers for the *Stevia* molecular research community and for breeding. Two types of markers, SSR (Simple Sequence Repeats) and SNP (Single Nucleotide Polymorphism) were identified using MISA (MIcroSAtellite) (http://pgrc.ipk-gatersleben.de/misa/misa.html) [26] and SOAPsnp (Short Oligonucleotide Analysis Package) software [27], respectively. In total, 10,070 SSRs were identified among the 80,160 unigenes (Additional file 3), accounting for 12.56% of all unigenes. Additionally, 1,136 unigenes contained more than 1 SSR. SSRs generally included 2 to 6 nucleotide repeat types and the number of repeats changed significantly among geneotypes. The mono-, di-, tri-, tetra-, penta- and hexanucleotide repeat SSRs in this study composed about 55.05%, 18.17%, 25.48%, 0.99%, 0.14% and 0.16% of the SSRs, respectively (Additional file 4: Figure S1). To facilitate the usage of the SSR markers as a resource for the *Stevia* molecular biology and breeding community, we designed primers for each of the SSRs using Primer3 (http://primer3.sourceforge.net/releases.php) (Additional file 5). Twenty primer pairs were randomly selected from the microsatellites. All 20 primer pairs had amplicons in 3 *Stevia* varieties (SR_1, SR_2 and SR_3), of which 5 primer pairs showed polymorphism and this results indicated that molecular markers could be used for marker-assisted breeding in *Stevia* (Additional file 4: Figure S2). As an alternative to SSRs, 44,510 SNP variations (in 11,000 unigenes) were also identified among the three genotypes (Additional files 6, 7 and 8). The high density SNP markers may be useful for molecular research of *Stevia* in the event that no SSR markers are available.

### Transcript profiling

**Figure 3:**
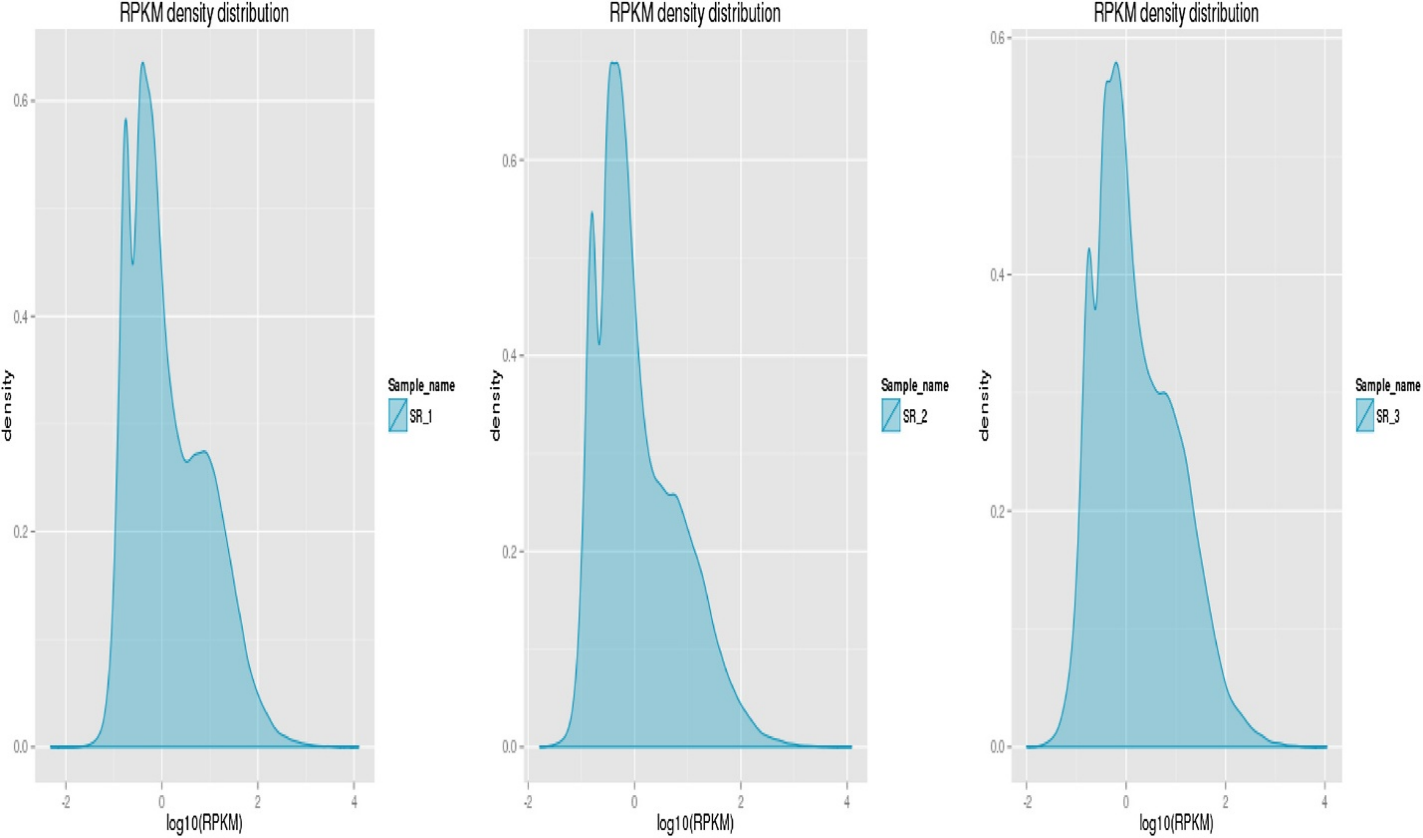
**Frequency distribution of SR-1, SR-2 and SR-3 by reads per kilobase per million (RPKM).**

The sensitivity of RNA-Seq facilitates the measurement of both the molar concentration and transcript length. We used the normalized-RPKM (reads per kilobase per million) to quantify the transcript level in reads, which facilitated the comparison of mRNA levels both within and between samples [28]. The three genotypes showed similar RPKM density distribution (Figure 3), which suggested that the transcript profiles of the three samples were similar. The RPKM density distribution of the three samples also showed that the transcripts were enriched at the RPKM region between 0.3 and 3.57; the percentages of transcripts in this region were 38.11%, 43.55% and 33.89% in SR-1, SR-2 and SR-3, respectively (Figure 3). We identified the most abundantly expressed unigenes in *Stevia* leaf because they were considered important for *Stevia* development. We focused on the top 2% (598 unigenes) most highly expressed genes of each sample (Additional file 9). The RPKM of those unigenes were greater than 156, 163 and 186 in SR-1, SR-2 and SR-3, respectively. Interestingly, the abundantly expressed unigenes in the three samples were enriched in metabolic pathways according to both KEGG and GO analysis. This suggested that the genes involved in metabolism be dominant in the three genotypes (Additional file 4: Figures S3 and S4), and many metabolism products, such as SGs, be presented in *Stevia*.

### Differentially expressed genes among three genotypes

**Figure 4:**
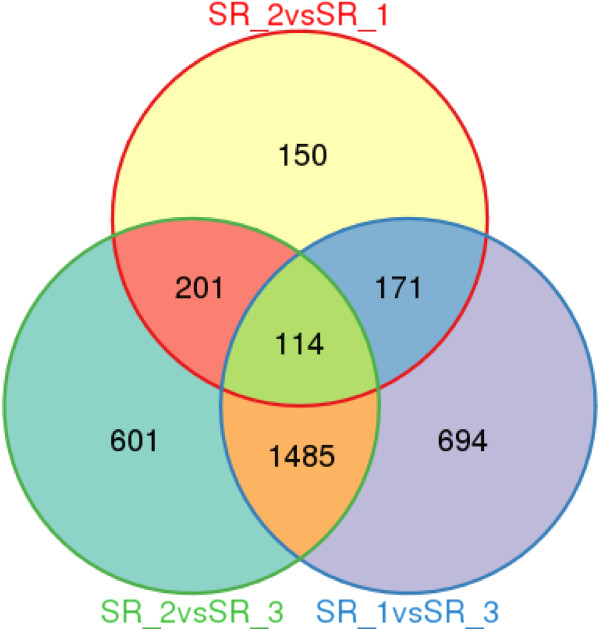
**Differential gene expression showed in Venn diagram form.**

The three genotypes (SR-1, SR-2 and SR-3) with significantly different amounts of ST and RA in their leaves (Table 1) had relatively higher similar transcript profiling (Figure 3). Consequently, we sought to analyze the differentially expressed unigenes in order to identify candidate genes involved in ST and RA biosynthesis. The DEGSeq program [29] was used to identify the differentially expressed unigenes among SR-1, SR-2 and SR-3. For SR-1 vs SR-2, 636 unigenes were differentially expressed, including 248 genes that were obviously up-regulated in SR-1 and 388 genes that were obviously down-regulated. For SR-1 vs SR-3, 2,464 unigenes were differentially expressed (1,185 up-regulated and 1,279 down-regulated in SR-1), and for SR-3 vs SR-2, 2,041 unigenes were differentially expressed (1,156 up-regulated and 1,246 down-regulated in SR-3). Among these differentially expressed genes, 114 genes were differentially expressed among all threes genotypes (Figure 4, Additional file 10).

To further analyze the possible function of unigenes with differential expression levels, we assessed their GO classifications. The 636 unigenes with differential expression between SR-1 and SR-2 were classified into 95 pathways by KEGG analysis, with clear enrichment in metabolic pathways (Additional file 4: Figure S5A). Because the amount of ST in SR-1 was significantly lower than SR-2 (Table 1), some of the 248 up-regulated unigenes of SR-1 vs SR-2 were likely to be directly or indirectly involved in ST biosynthesis. Similarly, because the amount of RA in SR-1 was significantly higher than SR-2, some of the 338 down-regulated unigenes were likely to participate in the RA biosynthetic pathway. In support of these data, both the down-regulated and up-regulated unigenes were enriched in metabolic pathways as determined by KEGG analysis (Additional file 4: Figure S5B and C). The unigenes of SR-3 vs SR-2 were also enriched in metabolic pathways by KEGG analysis (Additional file 4: Figure S6A). However, the occurrence of 2,041 unigenes with differential expressions between SR-3 and SR-2 suggested that other differences may contribute to the relatively larger number of differentially expressed unigenes. The down-regulated and up-regulated unigenes of SR-3 vs SR-2 were enriched in the biosynthesis of secondary metabolites and metabolic pathways, consistent with roles in ST and RA biosynthesis, respectively (Additional file 4: Figure S6B and C). In total, 315 overlapping unigenes were similar between the two comparisons (Figure 4, Additional file 11), which might narrow down the identification of genes that directly participate in the RA and ST biosynthetic pathway. As an example, one of the 315 unigenes (comp68371_c0; predicted as UGT76G1) is reported to be involved in the SG biosynthetic pathway. Additionally, two cytochrome P450s (comp57120_c0 and comp70800_c1), beta-1,3-glucanase (comp67196_c0) and beta-1,4-xylosidase (comp32324_c0) are good candidates for the SG biosynthetic pathway because they have all been reported to participate in the biosynthesis of SG precursors [30].

Additionally, because the RA and ST amounts in SR-1 and SR-3 were similar, the overlapping unigenes of SR-1 vs SR-3 might also be helpful for excluding unigenes that were not significantly associated with the RA and ST biosynthetic pathways. We found 114 overlapping unigenes with SR-1 vs SR-3. Exclusion of these 114 unigenes from the 315 overlapping unigenes for SR-1 vs SR-2 and SR-3 vs SR-2 left 201 remaining unigenes (Additional file 11), which were much more likely to be involved in the ST and RA biosynthetic pathways. This gene set included comp56279_c0 and comp57120_c0, which were predicted to encode UDP-glucuronosyltransferase and cytochrome P450 mono-oxygenase, respectively.

### The expression pattern of genes involved in the SG biosynthetic pathway

Seventeen steps catalyzed by various enzymes have been identified in the SG biosynthetic pathway [2, 31]. The initial seven steps synthesizing isoprenoids are shared with the MEP (methyl erythritol-4-phosphate) pathway; the next five steps are similar to the GA (gibberellic acid) biosynthetic pathway; and the remaining five steps are specific for the SG biosynthetic pathway. Sixteen genes have been reported to be involved in the 17 steps, including eight genes (*DXS*, *DXR*, *MCT*, *CMK*, *MDS*, *HDS*, *HDR* and *IDI*) in the initial seven steps, four genes (*GGDPS*, *CPPS*, *KS* and *KO*) in the next five steps and four genes (*KAH*, *UGT85C2*, *UGT74G1* and *UGT76G1*) in the remaining five steps [2]. Using RNA-seq, we investigated these reported genes using the data from the three genotypes to obtain more information about the transcription of these genes. All of the reported genes were identified within the RNA-seq data, suggesting that they are all expressed. Two copies, comp51020 and comp68460, were found for the *HDR* gene (the 7^th^ step), and one copy (comp51020) had three alternative splicing isoforms, which were comp51020_c0, comp51020_c1 and comp51020_c2. Three copies (comp54604_c0, comp61604_c0 and comp66218_c0) were found for *KAH* (the 13^th^ step).

**Figure 5:**
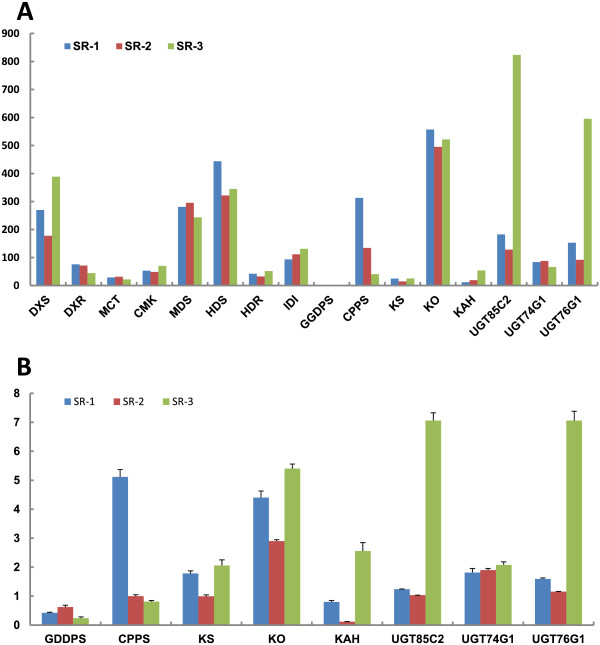
**Expression patterns of genes involved in steviol glycosides biosynthesis for three chemical types (SR-1, SR-2 and SR-3) by DGE and qPCR. (A)** Steviol glycosides biosynthesis genes detected by DGE. **(B)** Eight genes selected from above confirmed by qPCR. Results represent the means + standard deviation of triplicates.

We further investigated the expression levels of these genes among the three genotypes. *DXS*, *MDS*, *HDS*, *KO*, *UGT85C2* and *UGT76G1* were highly expressed in SR-1, SR-2 and SR-3 and showed obvious differences in expression among the three genotypes. *DXR*, *MCT*, *CMK*, *HDR*, *IDI*, *KS*, *KAH* and *UGT74G1* were relatively lowly expressed among the three genotypes (Figure 5A). Surprisely, the GGDPS gene, involved in the biosynthetic pathway of an SG precursor (geranylgeranyl diphosphate) was hardly detected in SR-1, SR-2 and SR-3 (Figure 5A). This might be due to the low expression of GGDPS among the three genotypes, which could not be detected by RNAseq. GDDPS was detected at very low levels by RT-qPCR, and the expressions of *CPPS*, *KS* and *KO* were similar to the RNAseq data (Figure 5B).

Because the last five of the 17 steps of the SG biosynthetic pathway were specific for *Stevia,* we next used RT-qPCR to investigate the expression pattern of the genes (*KAH*, *UGT85C2*, *UGT74G1* and *UGT76G1*), which are reported to be associated with the last five steps. Similar to the RNA-seq expression patterns, *KAH*, *UGT85C2* and *UGT76G1* were highest in the SR-3, compared to SR-1 and SR-2. The expressions of *KAH* and *UGT76G1* in SR-1 were obviously higher than that of SR-2 and SR-3, while *UGT74G1* was equally expressed in SR-1, SR-2 and SR-3 (Figure 5B), which was similar to the RNA-seq data. This verified that the RNA-seq in this study was reliable. Moreover, several reported genes known to be involved in SG synthesis had been identified in the differently expressed unigenes of SR-1 vs SR-2 and SR-3 vs SR-2, including *UGT76G1* (comp68371_c0) and *CPPS* (comp68805_c0). This further verified that it was feasible to use the RNA-seq approach to identify the genes associated with SG synthesis. Similarly, some triterpene biosynthetic genes from *Siraitia grosvenorii* had been found using RNA-seq and digital gene expression analysis [30].

### UGTs involved in SGs biosynthesis

Plant UDP-glycosyltransferases (UGTs) are a widely divergent group of enzymes that transfer a sugar residue from an activated donor to an acceptor molecule [32]. In *Stevia*, UGTs (such as *UGT74G1* and *UGT76G1*) were proposed to be involved in the production of SGs, which were unique in the plant world because of their intense sweetness and high concentration in the leaf tissue. In the study, we found 161 unigenes that were predicted to encode UDP-glycosyltransferases, including *UGT85C2*, *UGT74G1* and *UGT76G1,* which have been reported to be involved in the SG biosynthetic pathway (Additional file 12). There were 121 UGTs expressed in the three genotypes (RPKM >0). For each genotype, the expression of 141, 144 and 144 UGTs (RPKM >0) were detected in SR-1, SR-2 and SR-3, respectively (Additional file 12). Furthermore, one, two, and nine UGTs were specifically expressed in SR-1, SR-2 and SR-3, respectively (Red UTGs in Additional file 12). Based on the expression levels of those UGTs in SR-1, SR-2 and SR-3, we classified them into two groups (A and B). In group A, most UGTs in SR-3 were highly expressed, whereas, the UGTs of SR-1 and SR-2, except for a few UGTs, showed low expression. In contrast to the UGTs of SR-3 in group A, the UGTs in group B were expressed lowly. Most UGTs were highly expressed in SR-1 and SR-2, but about 1/3 of the UGTs were relatively lowly expressed in SR-2 (Additional file 4: Figure S7). The relationship between these UGTs and the diverse SGs needs to be studied further.

## Conclusion

We performed RNA-seq for three genotypes of *Stevia,* which had different RA and ST contents. In total, 80,160 unigenes were identified and classified into 250 pathways. A total of 10,070 SSRs and 44,510 SNPs were also identified. A total of 636, 2,464 and 2,041 unigenes showed differential expression in the comparison of SR-1 vs SR-2, SR-1 vs SR-3 and SR-2 vs SR-3. Moreover, the 315 unigenes that ped between the two comparisons of SR-1 vs SR-2 and SR-3 vs SR-2, were useful to identify the genes related to the SG biosynthetic pathway. Our study provided the first comprehensive report of the transcriptome of *Stevia* and provided a comprehensive resource for the research communities for *Stevia* or other closely related species. This study demonstrated the feasibility of using a combination of RNA-Seq and DGE to identify and study the genes involved in secondary metabolism for *Stevia*, a non-model herb plant. Moreover, candidate genes encoding enzymes potentially involved in SG biosynthesis could be rapidly identified by this approach.

## Methods

### Plant materials

Three different chemical types of *Stevia* with divergent SG composition from were selected in this study. Routinely, the *Stevia* leaves (3nd leaf from the top) were harvested and collected for transcriptome sequencing in the bud stage when the SGs peak. The plant leaves were then cut into small pieces and were immediately frozen in liquid nitrogen. All materials were stored at -80°C until further processing.

### RNA isolation and library preparation for transcriptome analysis

A total of 3 g RNA per sample was used as input material for the RNA sample preparations. All 3 samples had RIN values above 8.0. Sequencing libraries were generated using Illumina TruSeq™ RNA Sample Preparation Kit (Illumina, San Diego, USA) following manufacturer’s recommendations, and 3 index codes were added to attribute sequences to each sample. Briefly, mRNA was purified from total RNA using poly-T oligo-linked magnetic beads. Fragmentation was carried out using divalent cations under elevated temperature in Illumina proprietary fragmentation buffer. First strand cDNA was synthesized using random oligonucleotides and SuperScript II. Second strand cDNA synthesis was subsequently performed using DNA Polymerase I and RNase H. Remaining overhangs were converted into blunt ends via exonuclease/polymerase activities and enzymes were removed. After adenylation of 3′ ends of DNA fragments, Illumina PE adapter oligonucleotides were ligated to prepare for hybridization. To select cDNA fragments of preferentially 200 bp in length the library fragments were purified with AMPure XP system (Beckman Coulter, Beverly, USA). DNA fragments with ligated adaptor molecules on both ends were selectively enriched using Illumina PCR Primer Cocktail in a 10 cycle PCR reaction. Products were purified (AMPure XP system) and quantified using the Agilent high sensitivity DNA assay on the Agilent Bioanalyzer 2100 system. The clustering of the index-coded samples was performed on a cBot Cluster Generation System using TruSeq PE Cluster Kit v3-cBot-HS (Illumina) according to the manufacturer’s instructions. After cluster generation, the library preparations were sequenced on an Illumina Hiseq 2000 platform and 100 bp paired-end reads were generated.

### Analysis of Illumina sequencing results

The cDNA library was sequenced on the Illumina sequencing platform (GAII). Clean data (clean reads) were obtained by removing reads containing adapter, reads containing poly-N and low quality reads from raw data. At the same time, Q20, Q30, GC-content and sequence duplication levels of the clean data were calculated. All the downstream analyses were based on clean data with high quality. The left files (read1 files) from all libraries/samples were pooled into one big left.fq file, and right files (read2 files) into one big right.fq file. Transcriptome assembly was accomplished based on the left.fq and right.fq files using Trinity [20] with min_kmer_cov set to 2 and all other parameters set to default. Unigenes were used for BLAST searches with annotation against the NCBI Nr database (NCBI non-redundant sequence database) using an E-value cut-off of 10–5 (E-value <0.00001). After sequence assembly, the unigene sequences were also aligned by BLASTX to protein databases such as Swiss-Prot, KEGG and COG, in order to retrieve proteins with the highest sequence similarity to the given unigenes along with putative functional annotations. If results of different databases conflicted, then Swiss-prot database results were given precedence.

### Polymorphism detection

The MISA program (http://pgrc.ipk-gatersleben.de/misa/misa.html) was used to detect simple sequence repeats (SSRs) among sequences in MSGI 1.0. The minimum number of nucleotide repeats specified during SSR analysis was 20, 10, 7, 5, 5, and 5 for mono-, di-, tri-, tetra-, penta-, and hexanucleotide repeats, respectively. The maximum number of bases interrupting 2 SSRs in a compound microsatellite was set at 100 bp. Primers spanning each SSR were designed using the default parameter of the Primer3 program [33].

For SNP detection, clean reads were aligned to the reference transcriptome using SOAP2 [34], then duplicated reads and multi-mapped reads were filtered from the alignment results to eliminate the PCR interference and ambiguous mapping. SOAPsnp was used to call SNPs based on the sorted alignment results [35]. SNPs qualified for the following standards were selected as the final SNP sets: quality score of not lower than 20 (in PHRED scale), and base distance between two SNPs of greater than 5.

### Digital gene expression analysis

For digital gene expression analysis, differential expression analysis of two samples was performed using the DEGseq (2010) [29] R package. P values were adjusted using q values [36]. q value <0.0 05 & |log2 (foldchange)| > 1 was set as the threshold for significantly differential expression. Go enrichment analysis of the differentially expressed genes (DEGs) was performed using the GOseq based Wallenius non-central hyper-geometric distribution [37], which can adjust for gene length bias in DEGs. To correct for selection bias in category testing, we employed the following three-step methodology: First, the genes that were significantly DEGs between conditions were identified. The GOseq method works with any procedure for identifying DEGs. Second, the likelihood of DEGs as a function of transcript length was quantified by fitting a monotonic function to DEGs versus transcript length. Finally, the DEGs versus length function was incorporated into the statistical test of each category’s significance. This final step took into account the lengths of the genes that make up each category. KEGG pathway enrichment analysis of the DEGs was done using KOBAS [38]. KOBAS is a standalone commandline program written in Python (2.3.4). It consists of three modules: kparser, blast2ko and pathfind. Kparser uses BioPython (1.3.0) and Martel (0.9.0) to parse the KO and KEGG GENES datasets. The parsed information was managed with SQLite, a small C library that implements a self-contained, embeddable and zero-configuration SQL database engine. Blast2ko automatically annotated a set of new sequences (in FASTA format) with KO functional terms. Pathfind identified both the frequent and the enriched pathways in a given set of sequences. It calculated the FDR value by invoking the GeneTS (2.3) [39] package of the R (2.00) language [40] through RPy, an interface from Python to R. KOBAS would run on most Linux systems, and executables were freely available at http://kobas.cbi.pku.edu.cn/home.do.

### Real-time quantitative RT-PCR (RT-qPCR) assay

The total RNA from leaves of three samples was isolated using Qiagen RNA plant mini kit with on column DNAse digestion (Qiagen). Two micrograms total RNA was used for reverse transcription by M-MLVRT (Promega) with oligo (dT18) primer, and 1 μL RT product diluted to 20 μL ddH2O was used as template, three technical replicates and three biological replicates were applied for each gene expression analysis. Six hundred nanograms total RNA was reverse transcribed using the Primescript RT reagent kit with gDNA eraser (TakaRa). The cDNA diluted to 200 ng/μL was used for the qPCR assay with each gene-specific primers and SsoFast EvaGreen supermix (Bio-Rad) on the Bio-Rad CFX96 real-time system. Reactions were performed at 95°C for 1 min, 40 cycles of 95°C for 10s, and 58°C for 30s. All primers for RT-qPCR are listed in additional file 13.

### Analysis of SGs by HPLC

The *Stevia* leaves were harvested and collected for transcriptome sequencing in the bud stage when the SGs peak, oven-dried, and powdered by using a grinder. The extraction method of glycosides was based on the published method [39]. Briefly, for each sample, leaf powder (1.00 g) was first extracted with 50 mL of 80°C distilled water for 3 h, with shaking once every hour. After that, the mixture was purified with 0.16 g of a mixture of FeSO_4_ and CaCl_2_ (5:3) and centrifuged at 10,000 g for 10 min, and the supernatant (30 mL) was diluted to 50 mL with distilled water. Finally, the diluted supernatant (2 mL) was filtered through a 1 μ m pore size filter for measurement. Analyses were carried out by HPLC (Agilent 1100, USA) using an Agilent carbohydrate column of APS (250 × 4.6 mm, Phenomenex) maintained at 30°C and the flow rate of 1.0 mL min^-1^. The mobile phase was acetonitrile/H_2_O (80:20). The UV detector was set to a wavelength of 210 nm. Each sample was assayed for 30 min. Identification and calculation of stevioside and rebaudio-side A were carried out according to the method published previously [41]. The total glycoside content was calculated as the sum of the contents of stevioside and rebaudioside A.

### Availability of supporting data

The data sets supporting the results of this article are included within the article and its additional files.

## Electronic supplementary material

Additional file 1: **GO classification of the unigenes expressed in** ***Stevia*** **leaf.** (XLSX 1 MB)

Additional file 2: **KEGG classification of the unigenes expressed in** ***Stevia*** **leaf.** (XLSX 109 KB)

Additional file 3: **SSR markers identified in the unigenes expressed in** ***Stevia*** **leaf.** (XLSX 455 KB)

Additional file 4: Figure S1: SSR density. **Figure S2.** Polymorphism of the primers (SSR1-3) in 3 Stevia accessions. **Figure S3.** Pathway enrichment by KEGG. A, B and C, statistics of pathway enrichment for SR-1, SR-2 and SR-3, respectively. **Figure S4.** Enriched GO terms. A, B and C, GO term enrichment for SR-1, SR-2 and SR-3, respectively. **Figure S5.** Pathway enrichment by KEGG. A, B and C, statistics of pathway enrichment for SR-1 vs SR-2, down and up enriched KEGG pathways of SR-1 vs SR-2, respectively. **Figure S6.** Pathway enrichment by KEGG. A, B and C, statistics of pathway enrichment for SR-3 vs SR-2, down and up enriched KEGG pathways of SR-3 vs SR-2, respectively. **Figure S7.** Heat map of genes expressed in the steviol glycosides biosynthesis process. A. UGTs in the comparison of SR-1 vs SR-2, B. UGTs in the comparison of SR-2 vs SR-3. (PPTX 1 MB)

Additional file 5: **Primers for SSR markers.** (XLSX 2 MB)

Additional file 6: **SNP markers identified in the unigenes expressed in** ***Stevia*** **leaf.** (XLSX 10 MB)

Additional file 7: **SNP markers identified in the unigenes expressed in** ***Stevia*** **leaf.** (XLSX 12 MB)

Additional file 8: **SNP markers identified in the unigenes expressed in** ***Stevia*** **leaf.** (XLSX 12 MB)

Additional file 9: **Top 2% of unigenes in SR-1, SR-2 and SR-3.** (XLSX 292 KB)

Additional file 10: **The differently expressed unigenes of SR-2 vs SR-1, SR-1 vs SR-3, and SR-2 vs SR-3.** (XLSX 376 KB)

Additional file 11: **The overlapping unigenes between SR-2 vs SR-1 and SR-2 vs SR-3, and the overlapping unigenes among SR-2 vs SR-1, SR-1 vs SR-3 and SR-2 vs SR-3.** (XLSX 3 MB)

Additional file 12: **The unigenes predicted as UGTs.** (XLSX 19 KB)

Additional file 13: **List of primers used for RT-QPCR.** (XLSX 10 KB)

## Abbreviations

DGE: Digital gene expression
KOBAS: KO-Based annotation system
MEP: 2-*C*methyl-D-erythritol-4-phosphate pathway
GO: Gene ontology
KEGG: Kyoto encyclopedia of genes and genomes
DXS: 1-deoxy-D-xylulose-5-phosphate synthase
DXR: 1-deoxy-D-xylulose-5-phosphate reductoisomerase
MCT: 2-C-methyl-D-erythritol 4-phosphate cytidylyltransferase
CMK: 4-diphosphocytidyl-2-C-methyl-D-erythritol kinase
MDS: 2-C-methyl-D-erythritol 2,4-cyclodiphosphate synthase
HDS: 4-hydroxy-3-methylbut-2-enyl diphosphate synthase
HDR: (E)-4-hydroxy-3-methylbut-2-enyl diphosphate reductase
IDI: Isopentenyl diphosphate isomerase
IPI: Isopentenyl-diphosphate delta-isomerase
GGDPS: Geranylgeranyl diphosphate synthase
CPPS: Copalyl diphosphate synthase
KS: Kaurene synthase
KO: Kaurene oxidase
KAH: Kaurenoic acid hydroxylase
CYP450: Cytochrome P450
UDPG: UDP-glucosyltransferase.
