# Supplementary material for: RNA-Seq for gene identification and transcript profiling of three Stevia rebaudiana genotypes
Source: BMC Genomics. 2014 Jul 7;15(1):571. doi: 10.1186/1471-2164-15-571 (PMC4108789; doi:10.1186/1471-2164-15-571)
Supplement: Supplementary file 4 — Additional file 4: Figure S1: SSR density. Figure S2. Polymorphism of the primers (SSR1-3) in 3 Stevia accessions. Figure S3. Pathway enrichment by KEGG. A, B and C, statistics of pathway enrichment for SR-1, SR-2 and SR-3, respectively. Figure S4. Enriched GO terms. A, B and C, GO term enrichment for SR-1, SR-2 and SR-3, respectively. Figure S5. Pathway enrichment by KEGG. A, B and C, statistics of pathway enrichment for SR-1 vs SR-2, down and up enriched KEGG pathways of SR-1 vs SR-2, respectively. Figure S6. Pathway enrichment by KEGG. A, B and C, statistics of pathway enrichment for SR-3 vs SR-2, down and up enriched KEGG pathways of SR-3 vs SR-2, respectively. Figure S7. Heat map of genes expressed in the steviol glycosides biosynthesis process. A. UGTs in the comparison of SR-1 vs SR-2, B. UGTs in the comparison of SR-2 vs SR-3. (PPTX 1 MB) [file 12864_2013_6267_MOESM4_ESM.pptx]

## Slide 1
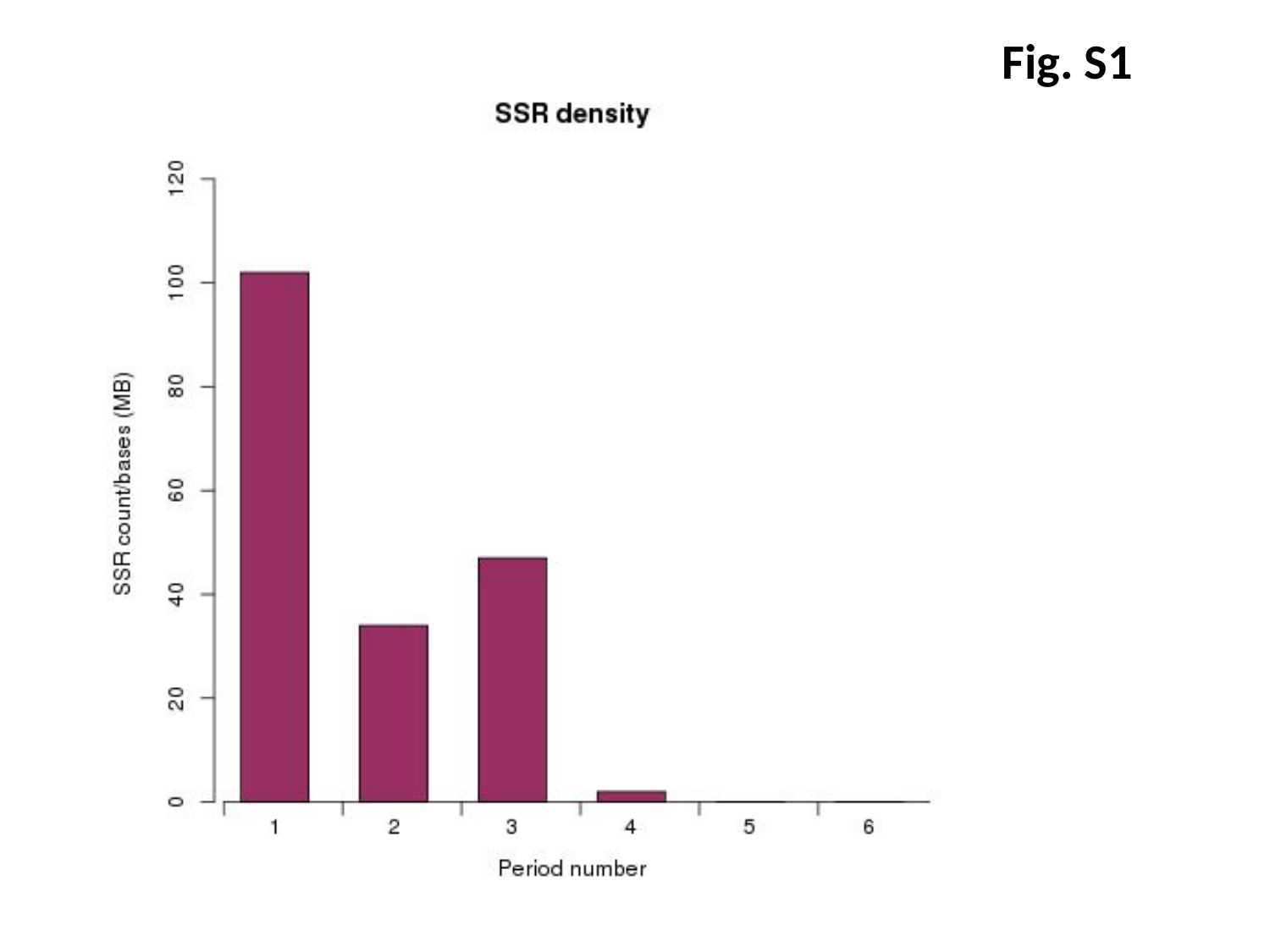

Fig. S1

## Slide 2
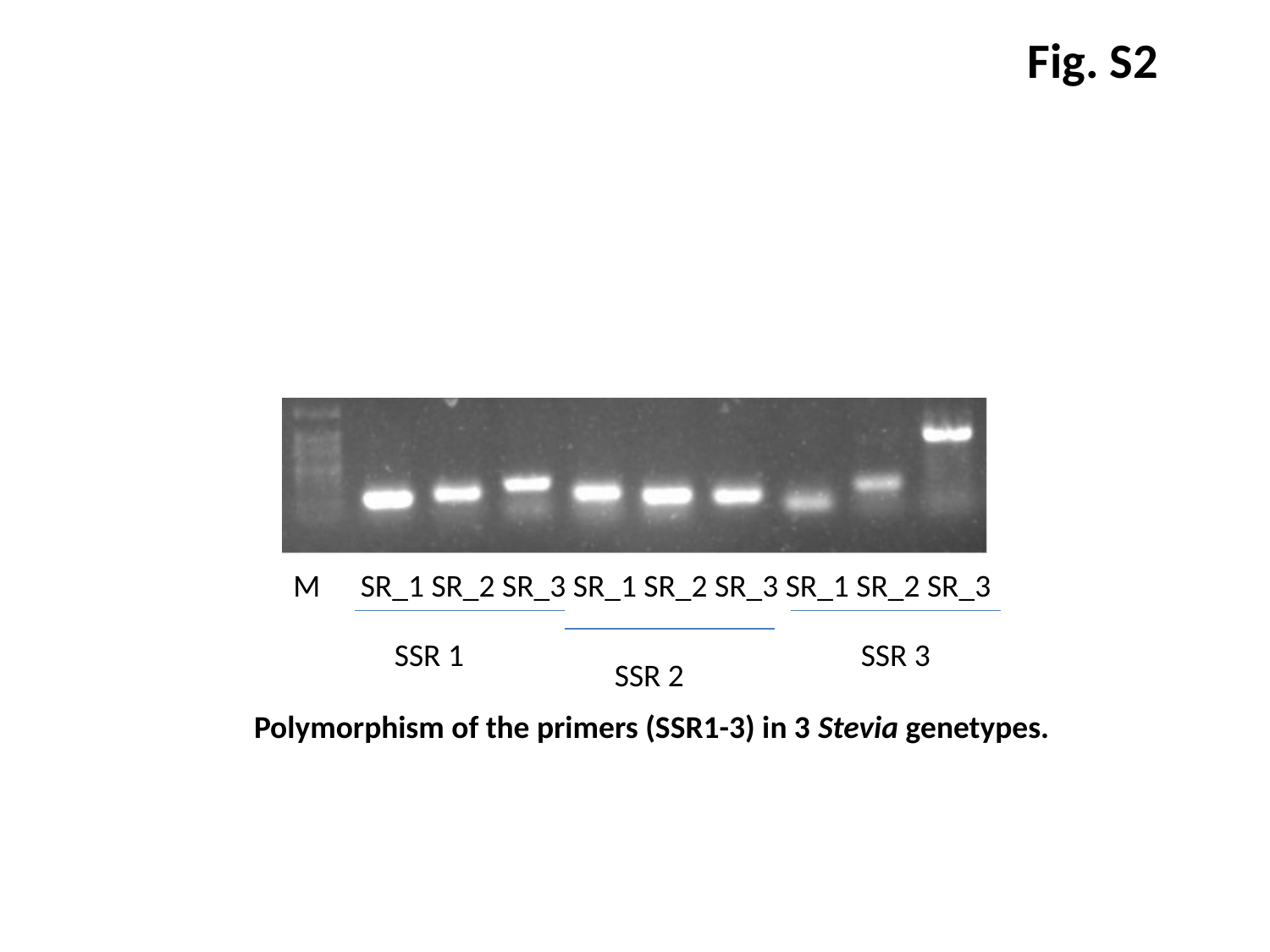

Fig. S2
M SR_1 SR_2 SR_3 SR_1 SR_2 SR_3 SR_1 SR_2 SR_3
SSR 1
SSR 3
SSR 2
Polymorphism of the primers (SSR1-3) in 3 Stevia genetypes.

## Slide 3
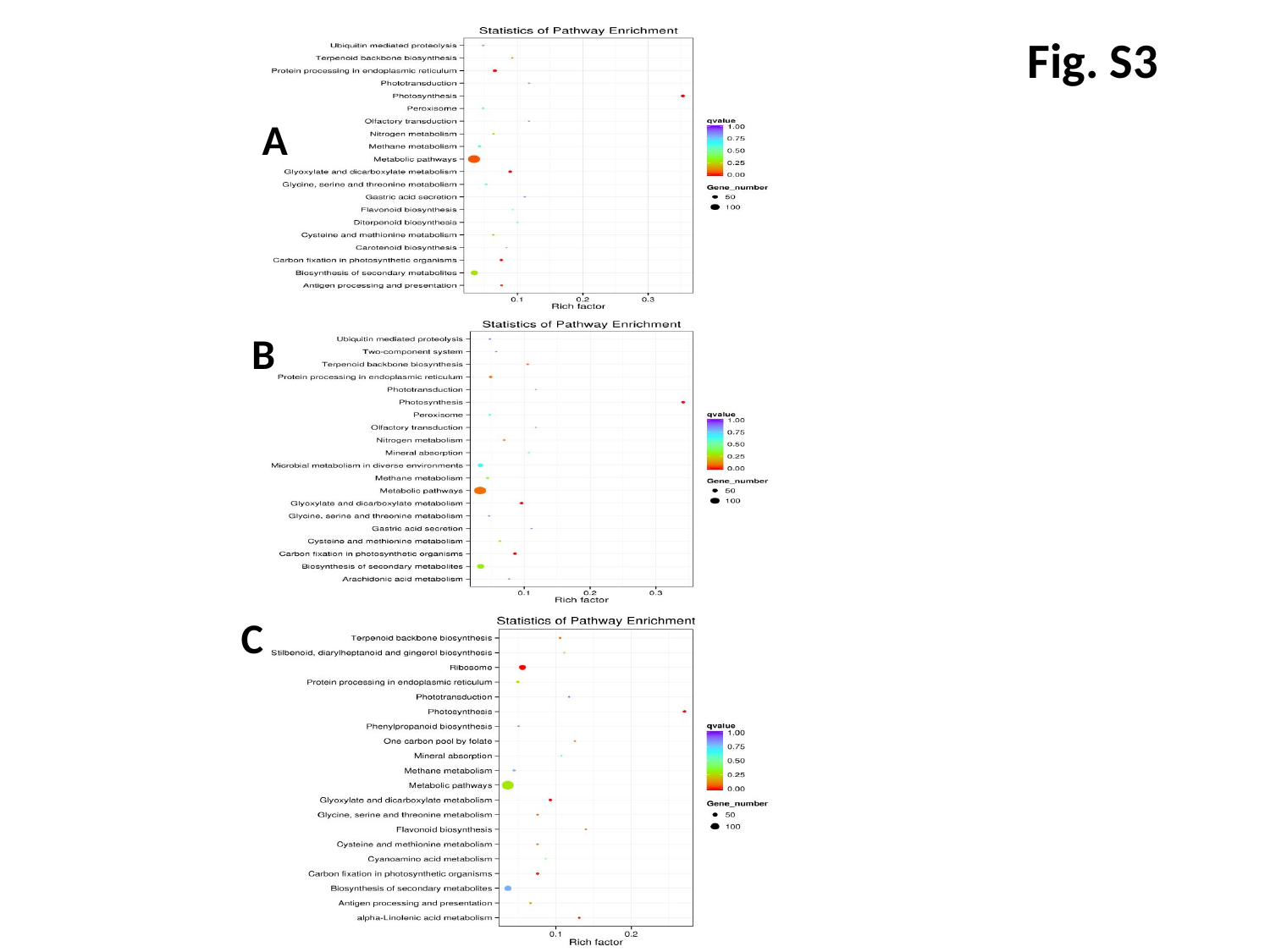

A
B
C
Fig. S3

## Slide 4
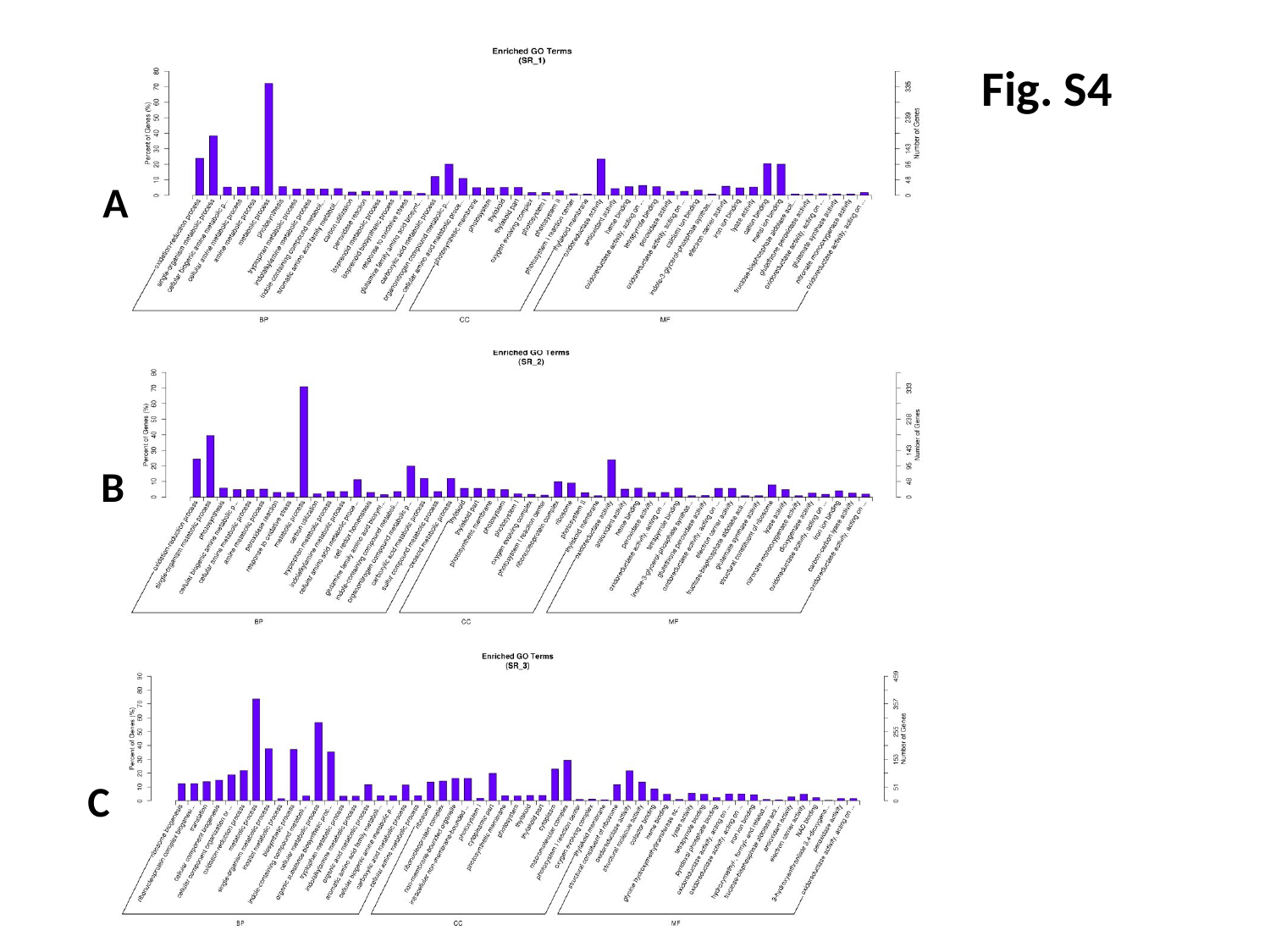

A
B
C
Fig. S4

## Slide 5
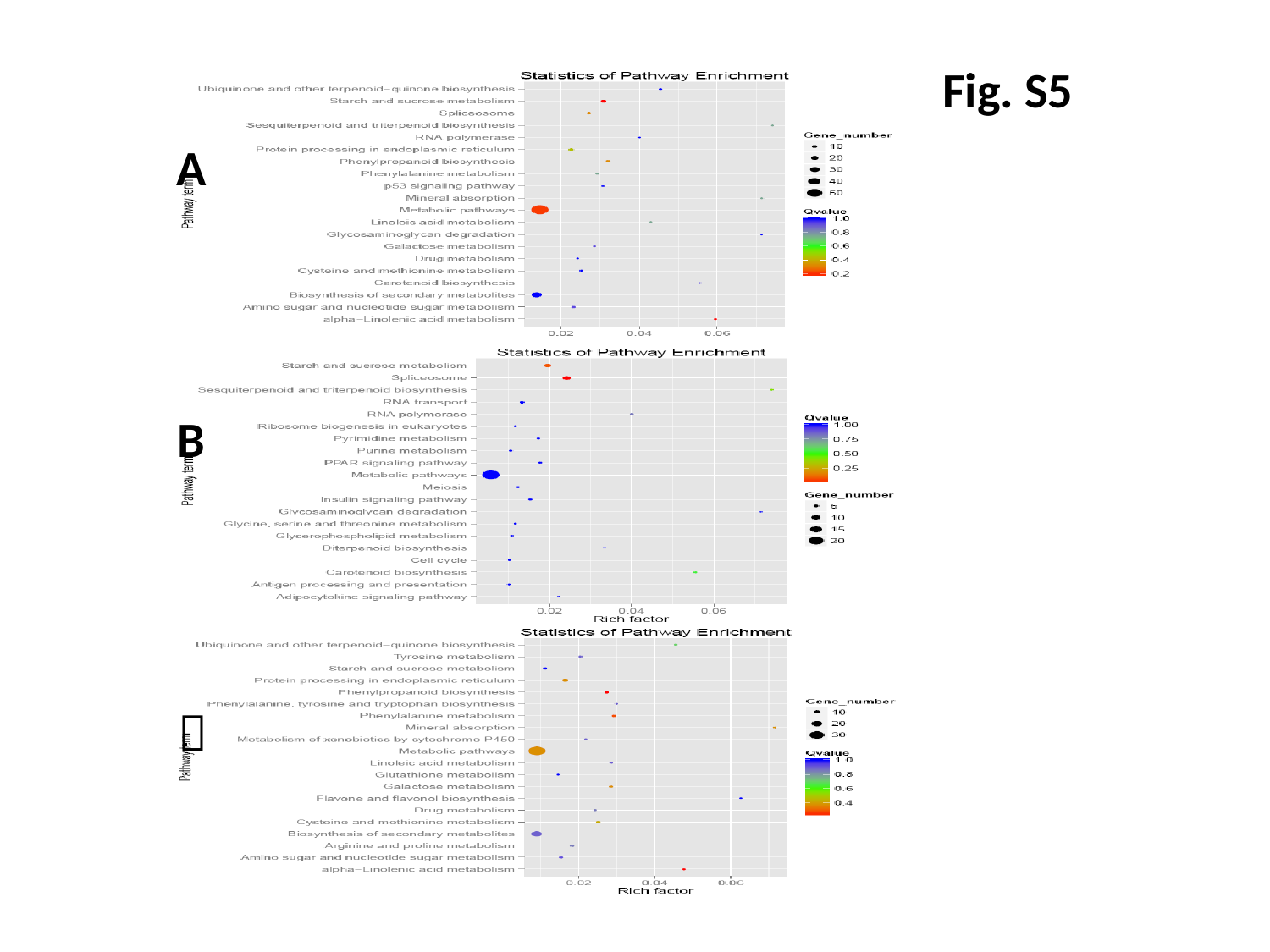

Fig. S5
A
B
Ｃ

## Slide 6
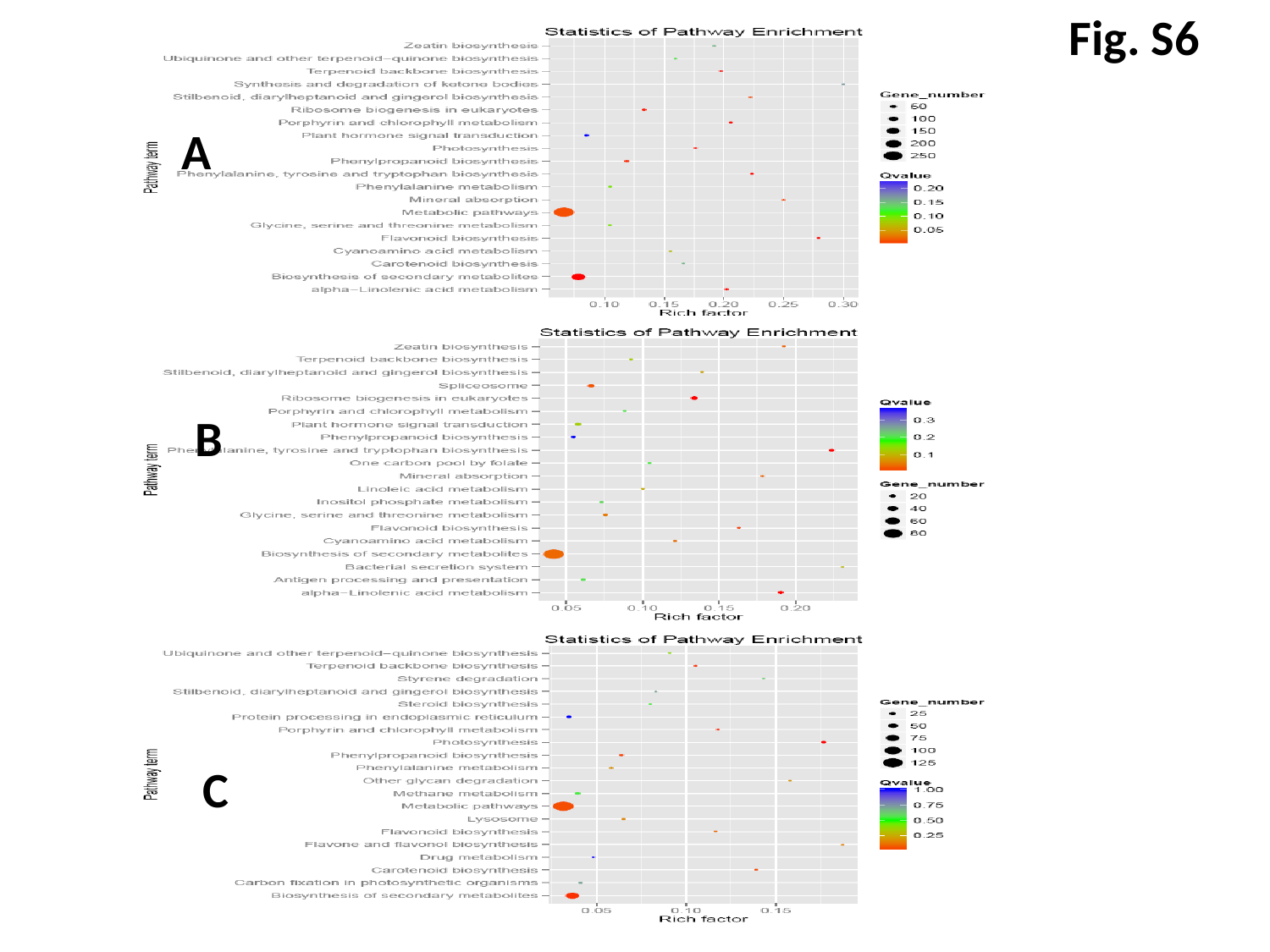

Fig. S6
A
B
C

## Slide 7
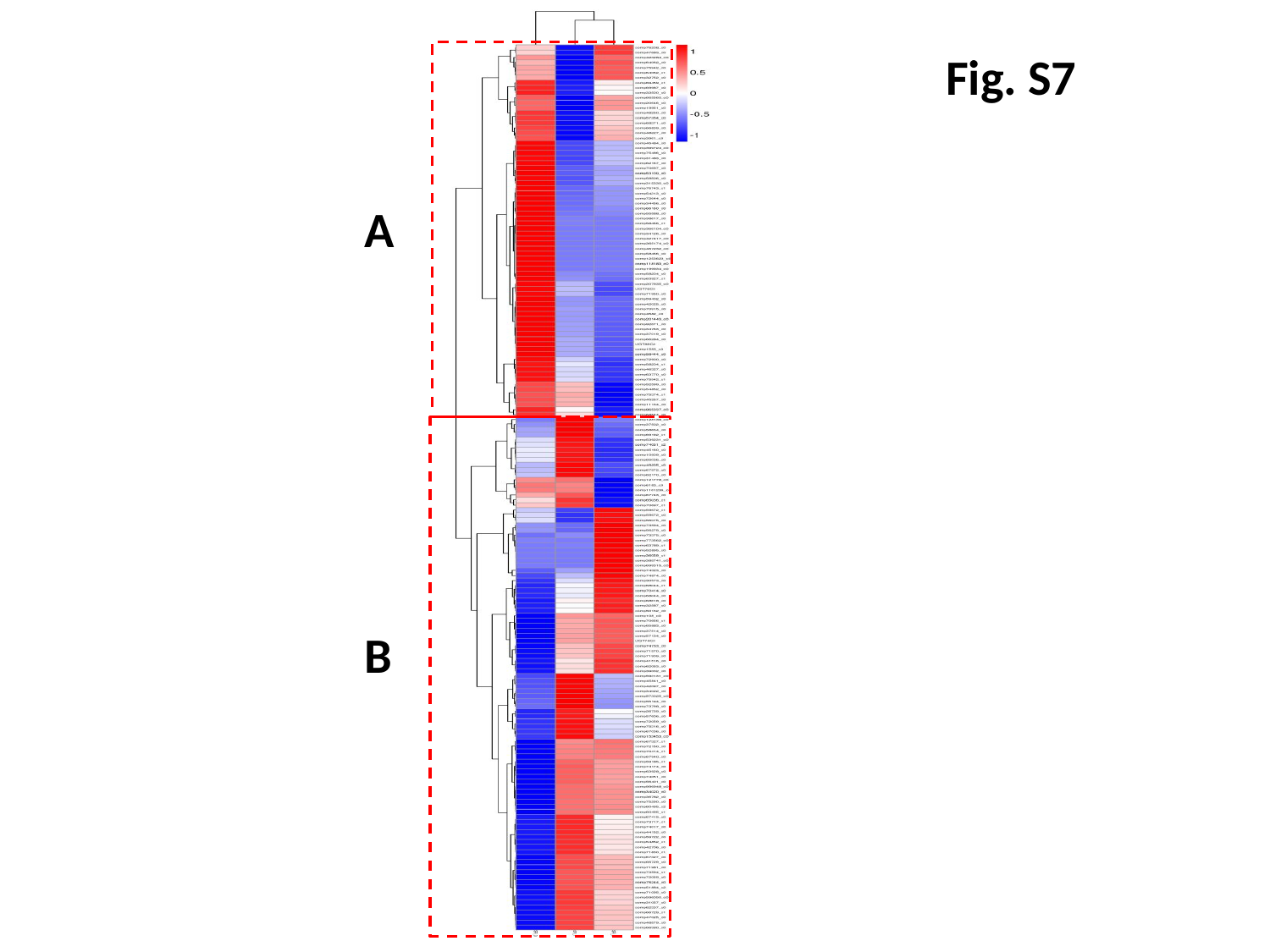

Fig. S7
A
B
